# Social attitude toward exotic salmonids aquaculture in the Beagle Channel, Argentina: Productive opportunity and/or conservation concerns?

**DOI:** 10.1017/cft.2026.10045

**Published:** 2026-09-09

**Authors:** Facundo Manuel Llompart, Javier Hernán Rojo, Silvina Alejandra Romano, Tomás Chalde

**Affiliations:** 1 Instituto de Limnologia Dr Raul A Ringuelet, Argentina; 2 Universidad Nacional de la Plata, Argentina; 3 Centro Austral de Investigaciones Cientificas, Argentina; 4 Universidad Nacional de Tierra del Fuego Antartida e Islas del Atlantico Sur, Argentina; 5 CONICET, Argentina; 6 Universidad Tecnologica Nacional Facultad Regional Rio Grande, Argentina

**Keywords:** aquaculture, policymakers, regulations, social perception, survey

## Abstract

The expansion of aquaculture often faces challenges related to environmental impacts and social acceptance, particularly when involving exotic species. In Argentina, where marine aquaculture remains marginal, recent proposals for salmonid farming in Tierra del Fuego province (TDF) have sparked public debate, balancing economic development against environmental concerns. In response, the local government enacted Law 1135/2021, banning salmonid farming in the province. This study explores public attitudes toward exotic salmonid aquaculture in TDF using a mixed-methods approach, combining press discourse analysis with an extensive online survey. Results indicate strong public resistance to net-pen salmonid farming in the Beagle Channel, especially among TDF residents and individuals linked to environmental sciences. In contrast, land-based aquaculture and farming of native species receive more support. Most respondents doubt the economic benefits of salmonid farming, and nearly 80% of TDF residents support the ban. Notably, while there is strong opposition to new exotic introductions, most respondents do not support eradication of established salmonid populations, which are widely accepted due to their long-standing presence and importance for recreational fishing. These findings reflect the nuanced views of the local community and emphasize the need for socially informed and transparent regulatory frameworks for aquaculture development in ecologically sensitive regions.

## Graphical abstract

**Figure:**
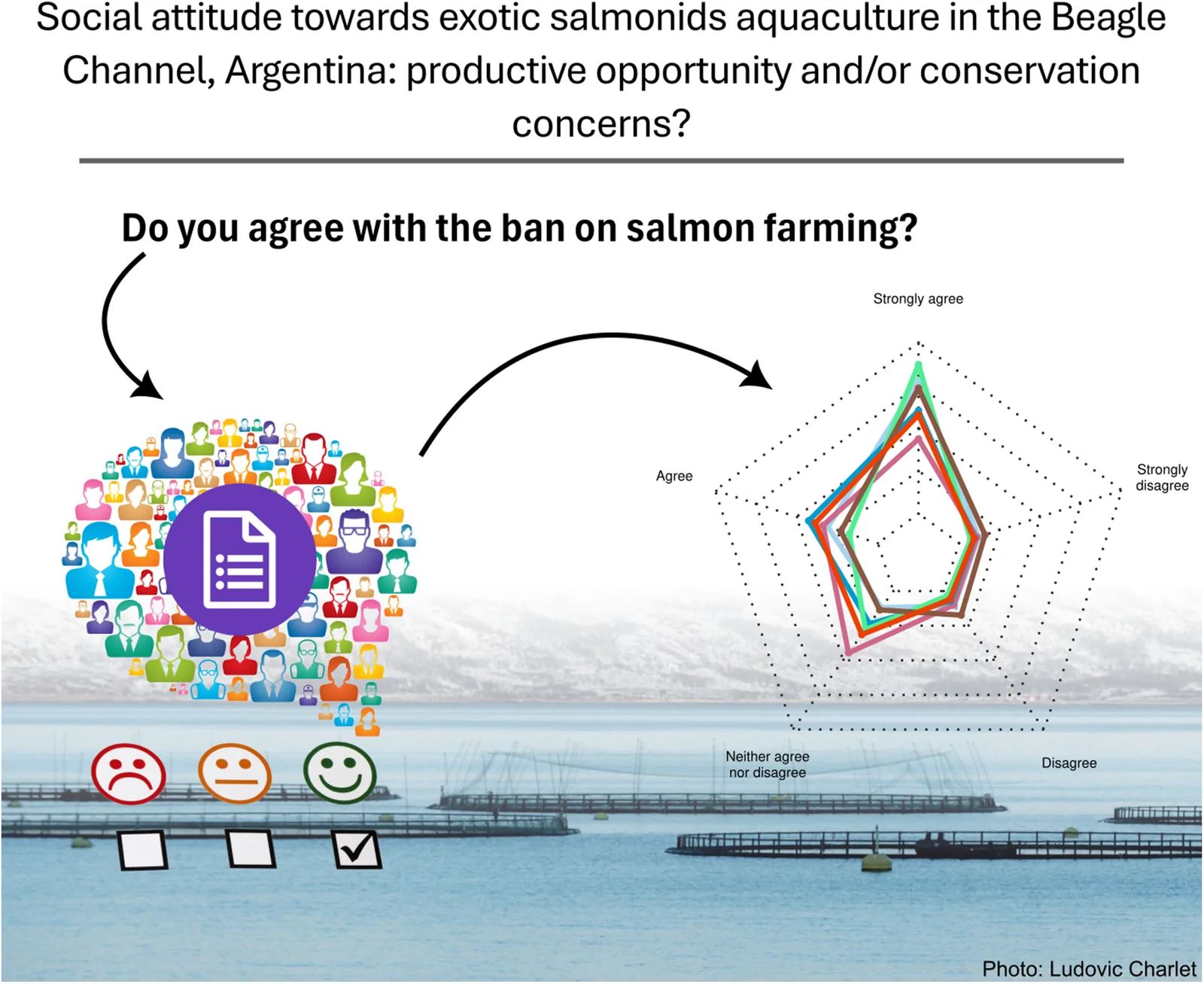

## Impact statement

Aquaculture is constantly expanding and requires urgent criteria to ensure sustainable practices and social acceptance, thus supporting the sector’s continued growth. Public opinion on trout and salmon aquaculture is diverse and often contradictory, conditioned by stakeholder values, trust in governance and access to information, making consensus difficult. This study explores public attitudes toward marine aquaculture of exotic salmonids on Tierra del Fuego Island, Argentina. Residents tend to show greater environmental concern due to place attachment (the emotional bond with their local environment), which may explain local resistance to aquaculture development. There is strong opposition to net-pen salmon aquaculture in the Beagle Channel, driven by concerns about ecological impacts, such as pollution, the escape of exotic species and effects on native biodiversity. However, opposition to terrestrial aquaculture systems is decreasing, indicating a preference for methods perceived as less harmful to the environment. The conflict surrounding salmonid aquaculture in the region reflects a complex social and environmental problem, involving scientific uncertainty, divergent stakeholder values and competing visions of regional identity and sustainability. Effective governance of salmonid aquaculture requires flexible and participatory approaches that integrate local knowledge, cultural values and legitimate concerns, beyond purely technical or economic considerations. Top-down promotion of aquaculture, which prioritizes economic growth without addressing social dimensions, risks generating local resistance, reputational damage and the erosion of local environmental values. The insights gathered in this study can help decision-makers promote sustainable aquaculture, considering public perceptions.

## Introduction

Aquaculture encompasses a variety of techniques and systems for cultivating aquatic organisms during all or part of their life cycle. Together, fisheries and aquaculture are vital to global food security, supplying approximately 90 million and 88 million tons of food in 2020, respectively, excluding algae (FAO, 2022). While global fish catches have plateaued since the 1990s, world aquaculture production increased by nearly 180% between 2000 and 2019 (Verdegem et al., 2023). In this context of global expansion, the development of criteria to ensure sustainable practices and social acceptability is crucial to support the continued growth of the sector (Falconer et al., 2023; Naylor et al., 2023). As with any productive activity, aquaculture generates economic and social benefits but may also result in negative externalities, such as the introduction of non-native species, deterioration of water quality, accumulation of plastic waste, excessive use of antibiotics and genetic homogenization of cultured stocks (Ahmed et al., 2019; Asche et al., 2022). In addition to these consequences directly linked to the ecosystem, there are other externalities as the visual impact that marine aquaculture facilities have on tourism (Perles-Ribes et al., 2022; Alsaleh and Wang, 2025). In this sense, it is known that visual impact represents a significant constraint in policy decisions related to the allocation of marine areas for aquaculture development in many countries (Campolunghi et al., 2025).Therefore, it is essential to have clear regulatory frameworks and effective monitoring mechanisms, given that the main barriers to the development of aquaculture globally encompass environmental, economic, social and cultural concerns (Alexander et al., 2015).

Over the past decade, trust in the regulatory frameworks, audits and overall oversight of aquaculture management has diminished in several communities and industry sectors (Condie et al., 2022). This mistrust may be more pronounced in countries where aquaculture is not well established and where public awareness of its potential benefits and risks is limited (Froehlich et al., 2017). In Argentina, aquaculture remains incipient, with a maximum annual production of only 6,022 tons recorded in 2022, primarily from freshwater species, while marine aquaculture has historically played a marginal role. The country’s first national aquaculture law, enacted in 2015, sought to foster the development of the sector. Under this framework, the national government promoted two marine aquaculture initiatives in the southern province of Tierra del Fuego A.I.A.S. (TDF), both focused on exotic salmonids (salmon and trout). The first project proposed industrial-scale production of Atlantic salmon (*Salmo salar*) using traditional net-pen. This initiative was supported through public funding to assess the feasibility of operations in the Beagle Channel (BC) and on the southern Atlantic coast of TDF, with the intention of eventual private sector development. The TDF government also signed a Cooperation Agreement supported by an international agency to determine the carrying capacity and site suitability for salmon aquaculture in the BC. The second project was part of Argentina’s national innovation strategy,^1^ ^,^^2^ and focused on developing Integrated Multi-Trophic Aquaculture (IMTA), combining the cultivation of rainbow trout (*Oncorhynchus mykiss*), blue mussel (*Mytilus chilensis*) and giant kelp (*Macrocystis pyrifera*) within the BC on a pilot-experimental scale.

Salmonids are among the most widely farmed finfish species globally, accounting for about 40% of the total biomass produced in marine aquaculture in 2020 (Cai et al., 2019; FAO, 2022). Beyond their commercial aquaculture significance, salmonids are also highly valued in recreational fisheries, generating substantial revenue not only in their native Northern Hemisphere (Whelan et al., 2006; Hughes, 2015) but also in regions like Argentina and Chile, where they were introduced in the early 20th century (Arismendi and Nahuelhual, 2007; Casalinuovo et al., 2017). However, these introductions have had significant ecological consequences, including predation and competition with native species (Arismendi et al., 2009; Soto et al., 2023), prompting a broader societal debate over the risks and benefits associated with the exploitation of exotic salmonids in South America (Gajardo and Laikre, 2003; Pascual et al., 2009; García de Leaniz et al., 2010; Macchi and Vigliano, 2014). In Argentina, the proposed development of net-pen aquaculture for exotic salmon and trout in the BC intensified tensions among civil society, government authorities and industry stakeholders that identify the natural capital as a distinguished element both in terms of landscapes and a differentiating element for tourist practice (Vereda, 2008; Barrios, 2024). It could also have a significant impact on tourism, as Ushuaia is one of the most important international tourist destinations in Argentina, particularly for nature tourism (Borla and Vereda, 2012). Following strong public debate and social opposition, decision-makers enacted the Provincial Law “Prohibition of Cultivation and Production of Salmonids” in 2021 (Poder Legislativo de Tierra del Fuego, 2021). Article 1 of this law prohibits all salmonid aquaculture and production activities in both freshwater and marine environments of TDF, with the aim of protecting natural and genetic resources. Exceptions are made only for stocking programs carried out under the authority of the competent regulatory body. Article 2 acknowledges the existence of small-scale rainbow trout farming operations, capped at 50 tons per year, and mandates that any new projects involving rainbow, brown or brook trout must be accompanied by a comprehensive environmental impact assessment.

In societies with strong civic engagement in decision-making related to natural resource use, it is essential to understand public perceptions, the factors shaping these views and how communities evaluate the trade-offs between economic activities and environmental impacts (Valcuende Del Río and Ruiz-Ballesteros, 2019). In the case of industrial salmon aquaculture in the BC, García et al. (2020) identified two contrasting societal perspectives. One group views aquaculture as an opportunity to capitalize on natural resources for local employment and economic development. In contrast, others question the net benefits of such projects when considering potential environmental degradation, impacts on natural landscapes and negative externalities affecting other productive sectors such as tourism. Within this context, the present study examines how different social profiles perceive alternative aquaculture systems, including land-based, open and closed systems, monoculture and IMTA, as well as their views on the environmental impacts and economic significance of salmonids and their level of support for the recently enacted provincial prohibition law. We hypothesize that place of residence, level of knowledge about salmonids and type of employment are key factors explaining the diversity of opinions regarding the establishment of salmon aquaculture in the BC.

## Materials and methods

### Press corpus analysis

Press corpus analysis was deployed on news articles retrieved from two major national media outlets (*La Nación* and *Página/12*) and two local newspapers (*El Diario del Fin del Mundo* and *Diario Prensa*) covering the period between the first social demonstrations against this activity (April 2018) and the enactment of the law (August 2021). The Spanish keyword “*Salmonicultura*” (*i.e.*, salmonid aquaculture) was used for the search. A textual corpus was compiled, and a summary of the main lexical components was produced to highlight key elements in the media framing. The ALCESTE method (Reinert, 1986) was applied through the IRaMuTeQ software to conduct the analysis. This methodology is practical to analyze large volumes of news text, identify recurring themes, linguistic patterns and ideological biases that manual reading cannot detect (Reinert, 1986).

### Questionnaire development and data collection

To collect data on public perceptions concerning aquaculture initiatives in TDF and the presence of exotic salmonids, an online survey was conducted. This methodology was chosen because it allows for a larger sample size in less time, facilitates local, national and international outreach and simplifies data analysis (Oladokun et al., 2025). The survey was administered through the Google Forms platform between July 19 and September 8, 2021 (Supplementary Material S1). The survey was disseminated *via* social media platforms (Facebook and Instagram), university and scientific institution websites, personal mailing lists, online forum groups and radio broadcasts to maximize outreach and reach a broad audience. The questionnaire was structured into two main sections.

#### Part A: Identification of the interviewee’s profile

A set of questions was developed to determine whether respondents’ profiles influenced their attitudes toward salmonids. Respondents were first classified as residents or nonresidents based on their place of residence in relation to TDF. A second classification, profession, was made to distinguish between individuals closely related to the topic and those not directly involved. Participants were categorized as “close-related profession” if their occupation was linked to the natural sciences (*e.g.*, biology students, biologists, professionals in nature-oriented careers and environmental managers). Additionally, to determine whether prior knowledge influenced perception (Osmond et al., 2023), a set of five questions concerning salmonids was included to construct a *knowledge index* (Supplementary Material S1). Respondents were classified as having high knowledge if at least 60% (three or more) answers were correct, and low knowledge if answered fewer than 60% (two or fewer) correctly. Anonymity of all participants was ensured throughout the process.

#### Part B: Specific questions to retrieve attitude toward the topic

To identify potential differences in attitudes toward salmon and trout, all questions regarding salmonids were duplicated to distinguish between the two species. The survey consisted of 21 closed-ended questions organized into five thematic sections to gather opinions on (1) Aquaculture: Aquaculture practices involving salmonids and native species in TDF across various production systems; (2) Salmonids interactions: Ecological interactions between salmonids and other fish species, including their environmental impact; (3) Salmonids eradication: The implementation of salmonid eradication measures; (4) Salmonids economic impacts: The economic importance of salmonids for TDF; and (5) Prohibition law: The level of acceptance of Provincial Law 1355/21, which bans the aquaculture of salmonids.

All questions were formulated using a five-point Likert scale with the following levels as proposed by Batterton and Hale (2017): “strongly agree” (or “very positively”), agree (or positively), “neither agree nor disagree” (or “neither positive nor negatively”), “disagree” (or “negatively”) and “strongly disagree” (or “very negatively”).

### Data analysis

Questionnaire responses were converted into ordinal numeric variables with five levels: Strongly Agree or Very Positively = 5, Agree or Positively = 4, Neither Agree nor Disagree, Neither Positive nor Negatively or Neutral = 3, Disagree or Negatively = 2 and Strongly Disagree or Very Negatively = 1. Missing responses were retained as NA values. To assess overall differences in respondents’ perception profiles, a Gower dissimilarity matrix was calculated among all pairs of respondents. This coefficient was selected because it is appropriate for ordinal variables and can accommodate missing values. Ordinal variables were treated following the modification proposed by Podani (1999), as implemented in the `gowdis` function of the R package FD. Differences in multivariate perception profiles among respondent groups were tested using permutational multivariate analysis of variance (PERMANOVA, Anderson, 2008), based on the Gower dissimilarity matrix. The analysis used 9,999 permutations of residuals under a reduced model. Residence (resident *vs.* nonresident), profession relatedness to natural sciences (directly related *vs.* not directly related) and knowledge index (high *vs.* low) were included as fixed factors, together with all interactions. When significant interaction terms were detected, pairwise comparisons were performed within the relevant factor levels. Radar plots were used to illustrate response patterns across questionnaire sections according to respondent profiles. Multivariate analyses were conducted in PRIMER v7 with the PERMANOVA+ add-on (Clarke and Gorley, 2015).

## Results

### Press corpus

**Figure 1.**
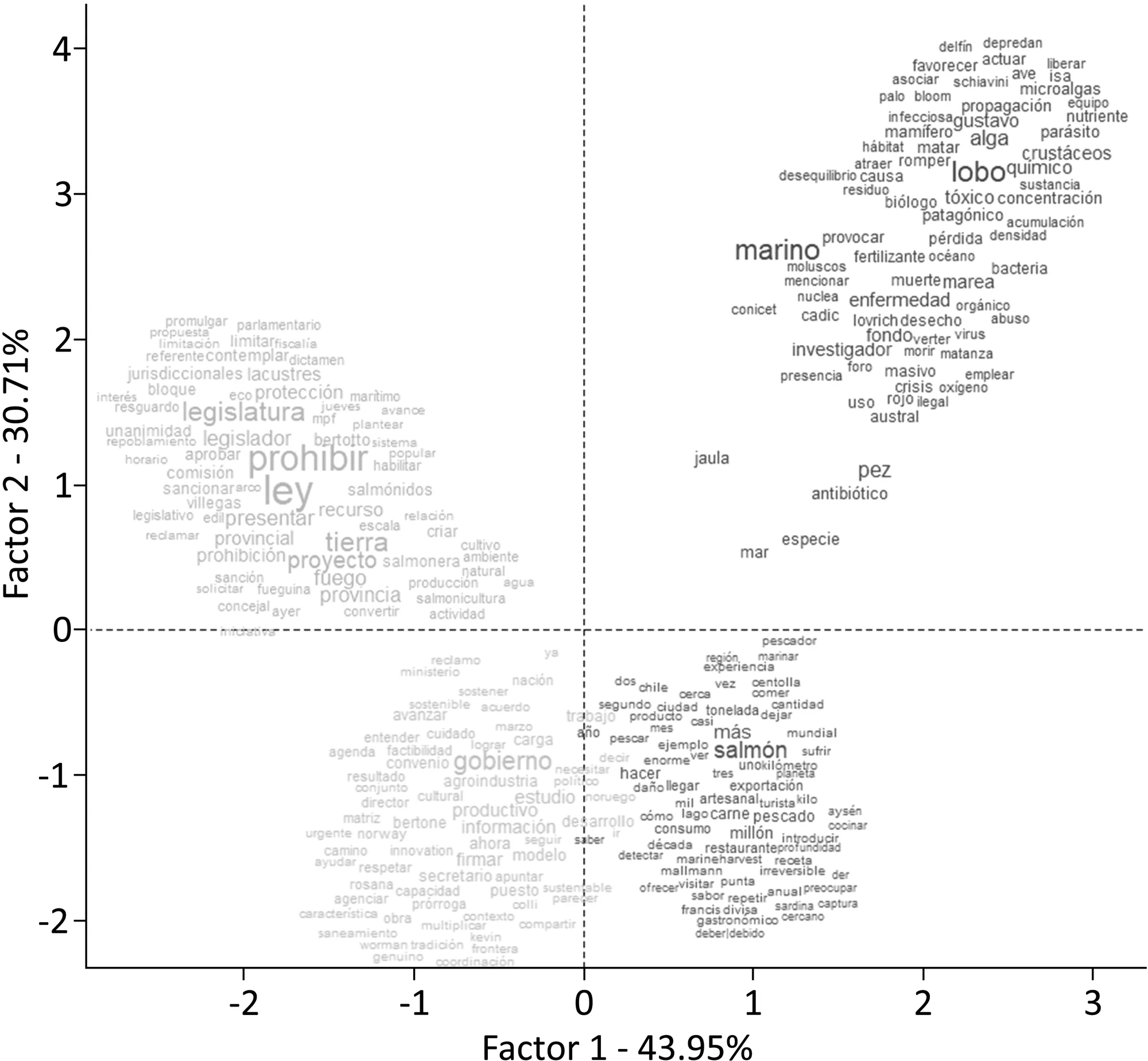
Lexical groups identified from the corpus related to salmon aquaculture and the distance between them. The words in English for each group can be found in Supplementary Material S2. Figure 1. long description.

A total of 13 media articles addressing salmon aquaculture in TDF were identified in the national press, while 32 were found in the local press, indicating a greater presence of the issue in local media outlets. The analysis of the literary corpus revealed four distinct lexical groups. Group 1 was associated with the legal prohibition of the activity (*e.g.*, prohibit, law, legislature); Group 2 pertained to themes of conservation and biodiversity; Group 3 reflected a narrative connected to the provincial government (*e.g.*, government, information); and Group 4 encompassed terminology linked to productive and economic aspects (*e.g.*, salmon, millions). Based on the proximity of the main terms in the plot, it was observed that the legal discourse (Group 1) was closely related to governmental themes (Group 3), which in turn were connected to economic productivity (Group 4). In contrast, the group associated with conservation and biodiversity (Group 2) appeared more isolated from the other clusters, suggesting a weaker integration of environmental discourse within the broader media narrative (Figure 1).

### Surveys analysis

A total of 492 surveys were collected over a 52-day period, with 68.1% corresponding to residents (*n* = 335) and 31.9% to nonresidents (*n* = 157) of TDF. Regarding educational attainment, 31.7% (*n* = 156) of respondents reported having completed university studies. The most represented age groups were 31–40 years (*n* = 146; 30%) and 41–50 years (*n* = 144; 29%). Multivariate response profiles differed significantly according to residence, profession relatedness to natural sciences and knowledge index (PERMANOVA, *p* < 0.01 in all cases; Table 1). Profession relatedness showed the strongest effect on overall perception patterns (Pseudo-F = 24.59, *p* = 0.0001), followed by knowledge index (Pseudo-F = 8.64, *p* = 0.002) and residence (Pseudo-F = 7.45, *p* = 0.003). Among interaction terms, only the interaction between profession relatedness and knowledge index was significant (Pseudo-F = 4.75, *p* = 0.0289), indicating that differences associated with knowledge level depended on whether respondents had a profession directly related to natural sciences or not. In contrast, the interactions Residence × Profession, Residence × Knowledge Index, and the three-way interaction were not significant (*p* > 0.05; Table 1). Pairwise comparisons within levels of profession relatedness showed that respondents with high and low knowledge index did not differ significantly within the group whose profession was directly related to natural sciences (*t* = 1.72, *p* = 0.0917), whereas significant differences were detected between high- and low-knowledge respondents within the group whose profession was not directly related to natural sciences (*t* = 3.63, *p* = 0.0001) (Table 2).

**Table 1.** PERMANOVA results for multivariate response profiles Table 1. long description.

Factors	df	MS	Pseudo-F	*p*(perm)	Unique perm
Residence (R)	1	0.20	7.45	0.003*	9,987
Profession (P)	1	0.66	24.59	0.0001	9,969
Index (I)	1	0.23	8.64	0.002	9,982
R x P	1	0.05	2.19	0.16	9,974
R x I	1	0.09	3.49	0.07	9,979
P x I	1	0.12	4.75	0.02*	9,966
R x P x I	1	0.06	2.46	0.12	9,969
Residual	484	0.02	—	—	—
Total	491	—	—	—	—

*Note:* The table shows degrees of freedom (df), mean squares (MS), Pseudo-F values, permutation-based p-values [p(perm)], the number of unique permutations and the student’s statistics (t), * p < 0.01.

**Table 2.** Results of pairwise comparisons for the significant interaction between profession relatedness and knowledge index Table 2. long description.

Group index	*t*	*p*(perm)	Unique perms
Within level “close” of factor “Profession”			
High, low	1.7221	0.0917	9,988
Within level “no-close” of factor “Profession”			
High, low	3.6334	0.0001	9,977

*Note:* Student’s t values, permutation-based p-values [p(perm)] and the number of unique permutations are shown.

#### Section 1: Aquaculture

**Figure 2.**
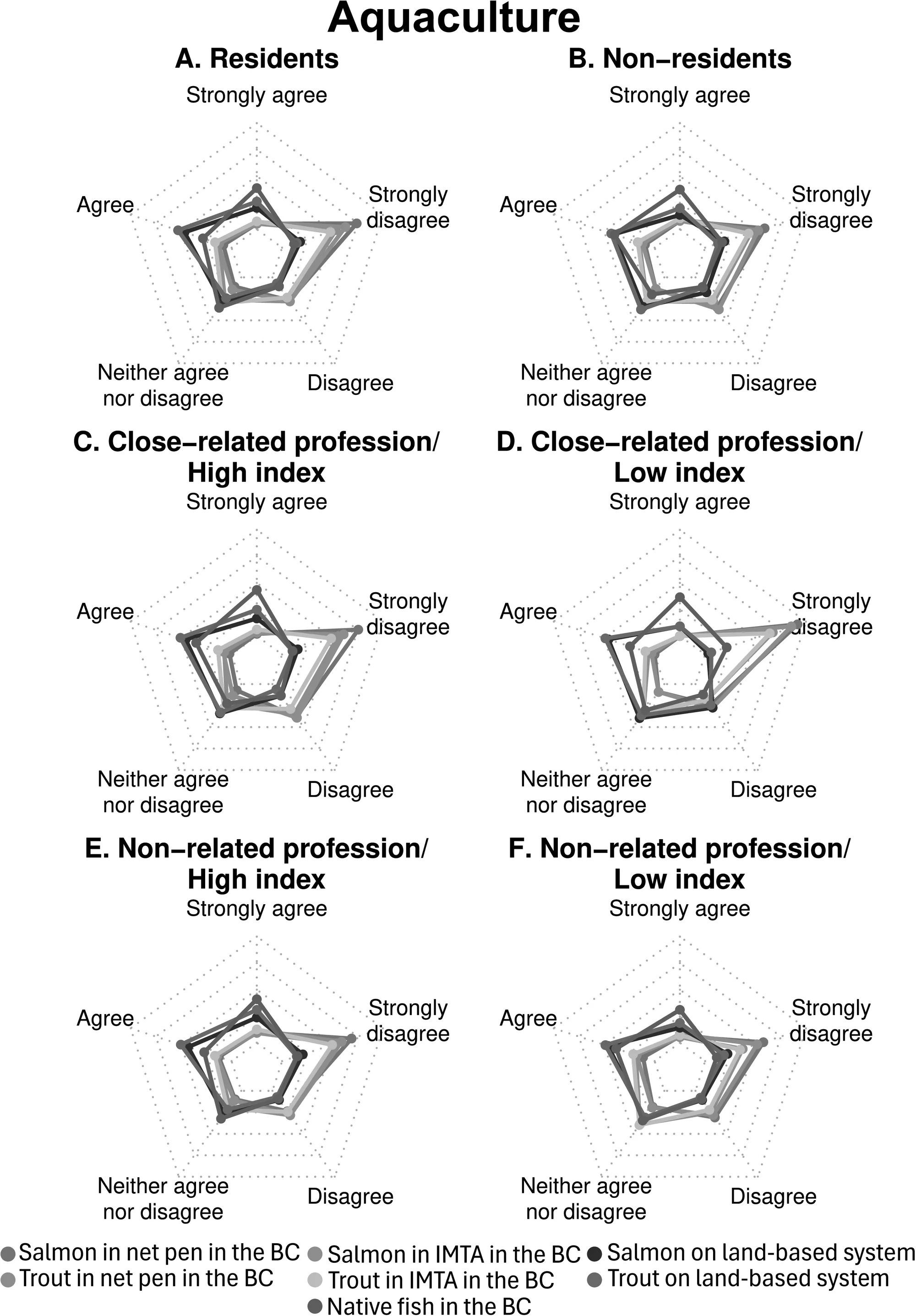
Radar chart displaying respondents’ perceptions across the questions included in Section 1: Aquaculture. A brief description of each question, along with its corresponding line color, is provided. BC, Beagle Channel; IMTA, Integrated Multi-Trophic Aquaculture. Figure 2. long description.

A consistent response pattern was observed across respondent profiles, with a marked tendency toward strong disagreement regarding open net-pen aquaculture of salmonid species in the BC, in contrast with IMTA systems (Figure 2). Salmon aquaculture was consistently perceived more negatively than trout, regardless of the production site (BC or land-based) or aquaculture system (IMTA or monoculture). These trends were more pronounced among residents (Figure 2A) than among nonresidents (Figure 2B). This overall pattern was broadly maintained across the four combinations of profession relatedness and knowledge index, although differences associated with knowledge level were slightly more apparent among respondents without a close-related profession (Figure 2E, F) than among those with a close-related profession (Figure 2C, D). Nonetheless, land-based aquaculture of any salmonid species was generally viewed as acceptable across profiles. Additionally, a substantial proportion of respondents strongly agreed with the idea of native fish aquaculture, despite the production modality not being specified.

#### Section 2: Salmonid interactions

**Figure 3.**
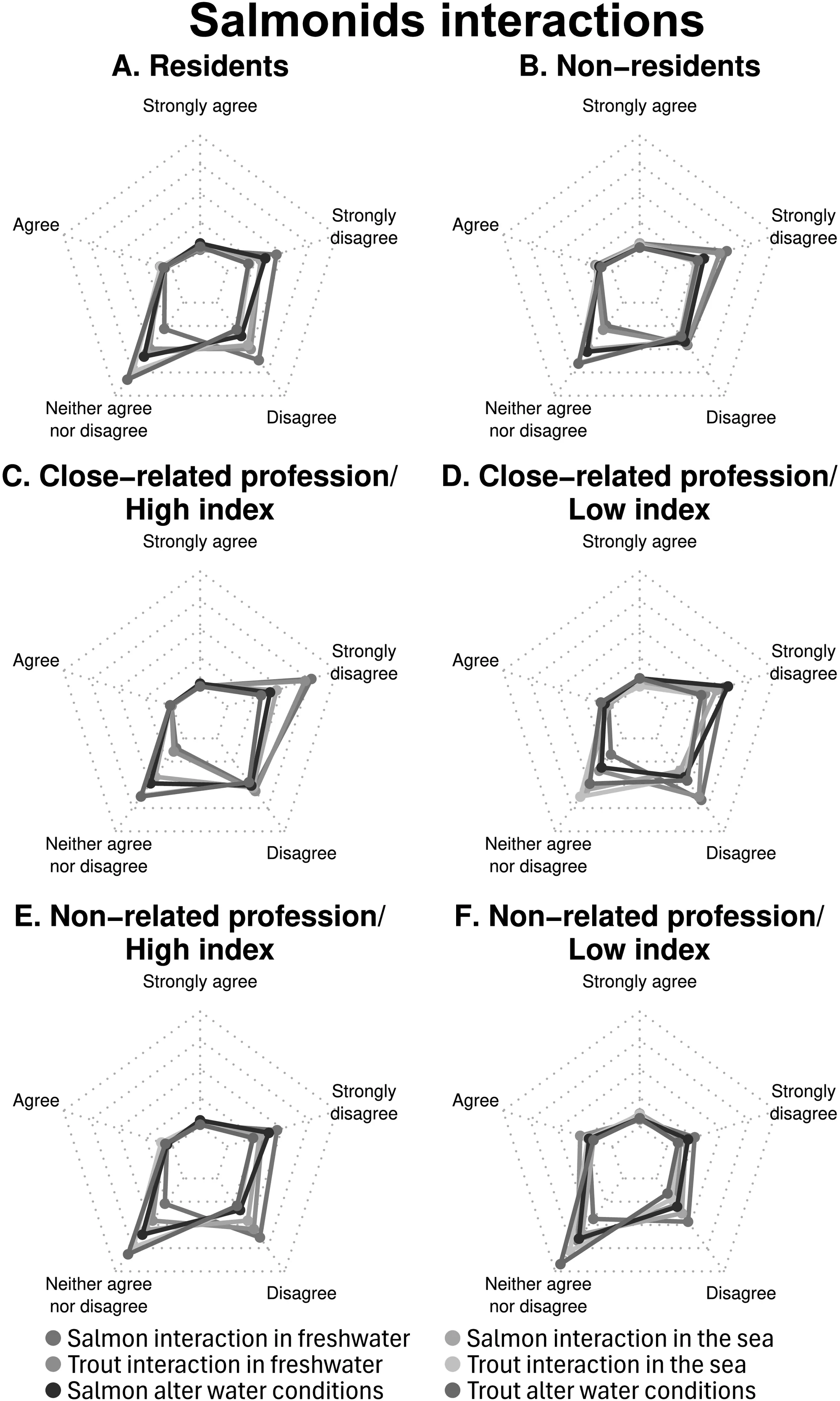
Radar chart displaying respondents’ perceptions across the questions included in Section 2: Salmonid interaction. A brief description of each question, along with its corresponding line color, is provided. Figure 3. long description.

Regarding ecological impacts, very few respondents reported perceiving any positive effects of salmonids on other fish species or the aquatic environment (Figure 3). Most indicated that trout exert neither positive nor negative effects. Moreover, nonresidents (Figure 3B) perceived that trout interact very negatively with the freshwater environment. Salmon were consistently viewed as having a greater ecological impact than trout. Respondents with a close-related profession (Figure 3C, D) generally expressed more negative perceptions of salmonid effects on native fish and water quality, while differences between high- and low-knowledge respondents were more evident among those without a close-related profession, particularly regarding perceived effects on water conditions (Figure 3E, F).

#### Section 3: Salmonid eradication

**Figure 4.**
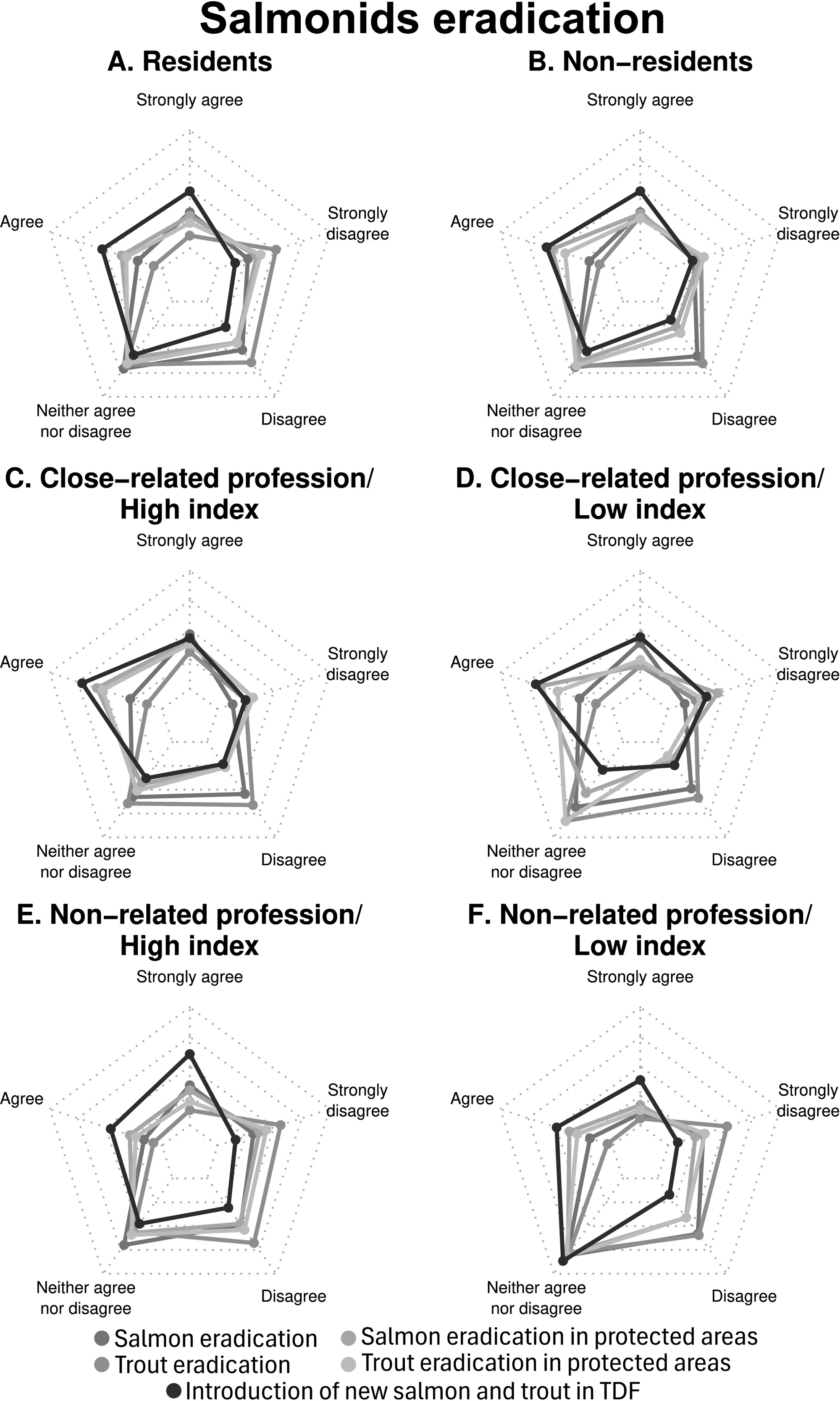
Radar chart displaying respondents’ perceptions across the questions included in Section 3: Salmonid eradication. A brief description of each question, along with its corresponding line color, is provided. Figure 4. long description.

No consistent response patterns were observed across or within respondent profiles for the questions in this section (Figure 4). Most residents expressed disagreement or strong disagreement with the eradication of trout (Figure 4A). Although some nonresidents shared this view, a high percentage were in favor of such action (Figure 4B). In contrast, “neutral” was the most frequently selected response concerning the eradication of salmon across all groups. Respondents without a close-related profession (Figure 4E, F) tended to oppose the complete eradication of trout, whereas those with a close-related profession (Figure 4C, D) more frequently selected “neutral” or “disagree.” Differences associated with knowledge level were also apparent for salmon eradication, with respondents with a high knowledge index (Figure 4C, E) tending to show greater support for this action than those with a low knowledge index (Figure 4D, F), who more often selected neutral responses. Regardless of respondent profile, no clear consensus was found regarding the potential eradication of salmonids exclusively from protected areas within TDF. Nevertheless, all respondent groups consistently indicated a willingness to accept the continued presence of already established salmonid populations.

#### Section 4: Salmonid economic impact

**Figure 5.**
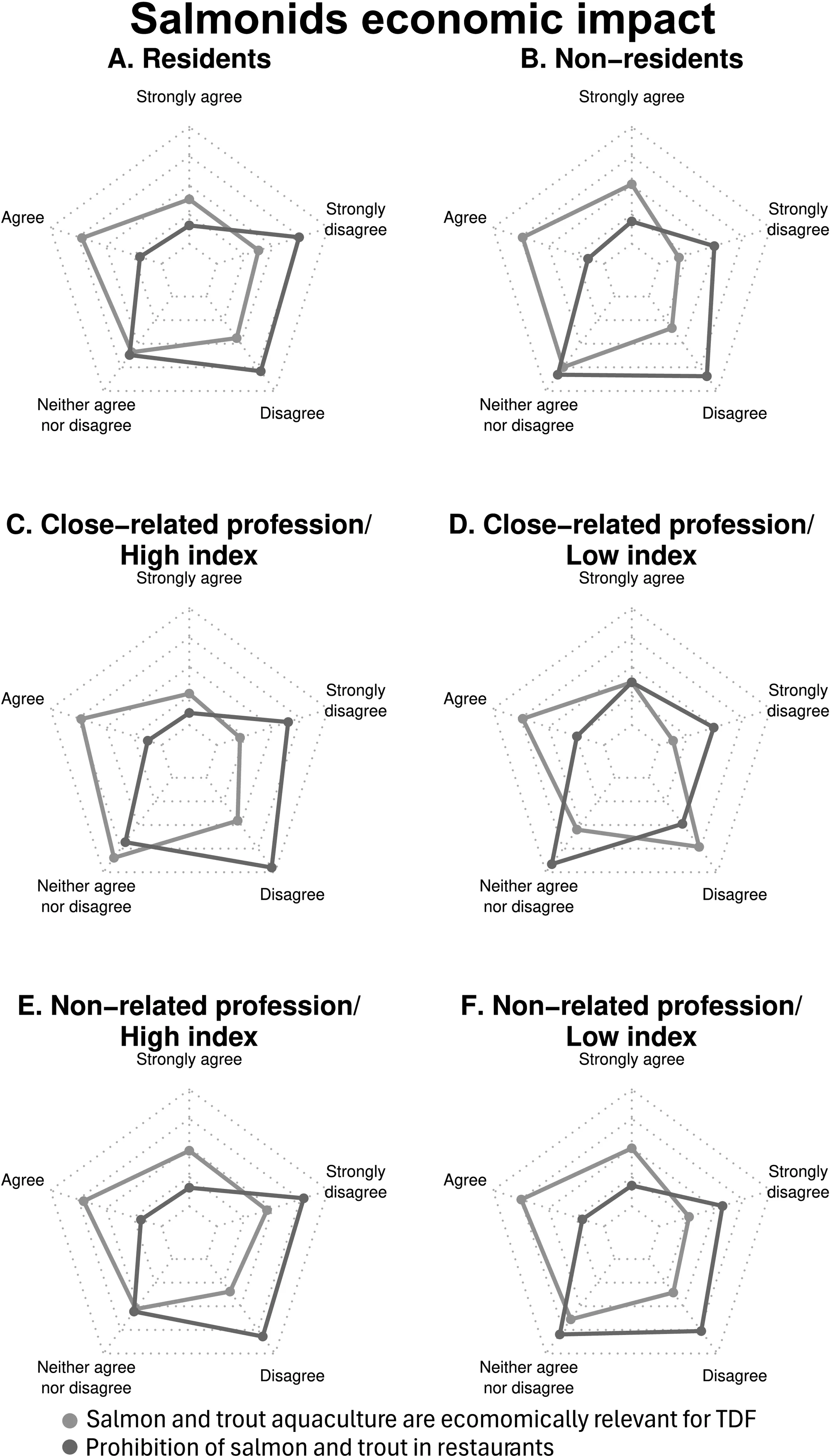
Radar chart displaying respondents’ perceptions across the questions included in Section 4: Salmonid economic impact. A brief description of each question, along with its corresponding line color, is provided. TDF, Tierra del Fuego A.I.A.S. Figure 5. long description.

With respect to the perceived economic implications of salmonids, residents and nonresidents showed a slight tendency to agree that salmon and trout aquaculture could represent a significant economic opportunity for TDF (Figure 5A, B). Likewise, the four combinations of profession relatedness and knowledge index (Figure 5C–F) showed a tendency to disagree or strongly disagree with the prohibition of human consumption of salmon and trout in the gastronomic sector. This disagreement tended to be stronger among respondents with a high knowledge index (Figure 5C, E), whereas perceptions of aquaculture as an economic opportunity for TDF remained comparatively similar across combinations.

#### Section 5: Prohibition law

**Figure 6.**
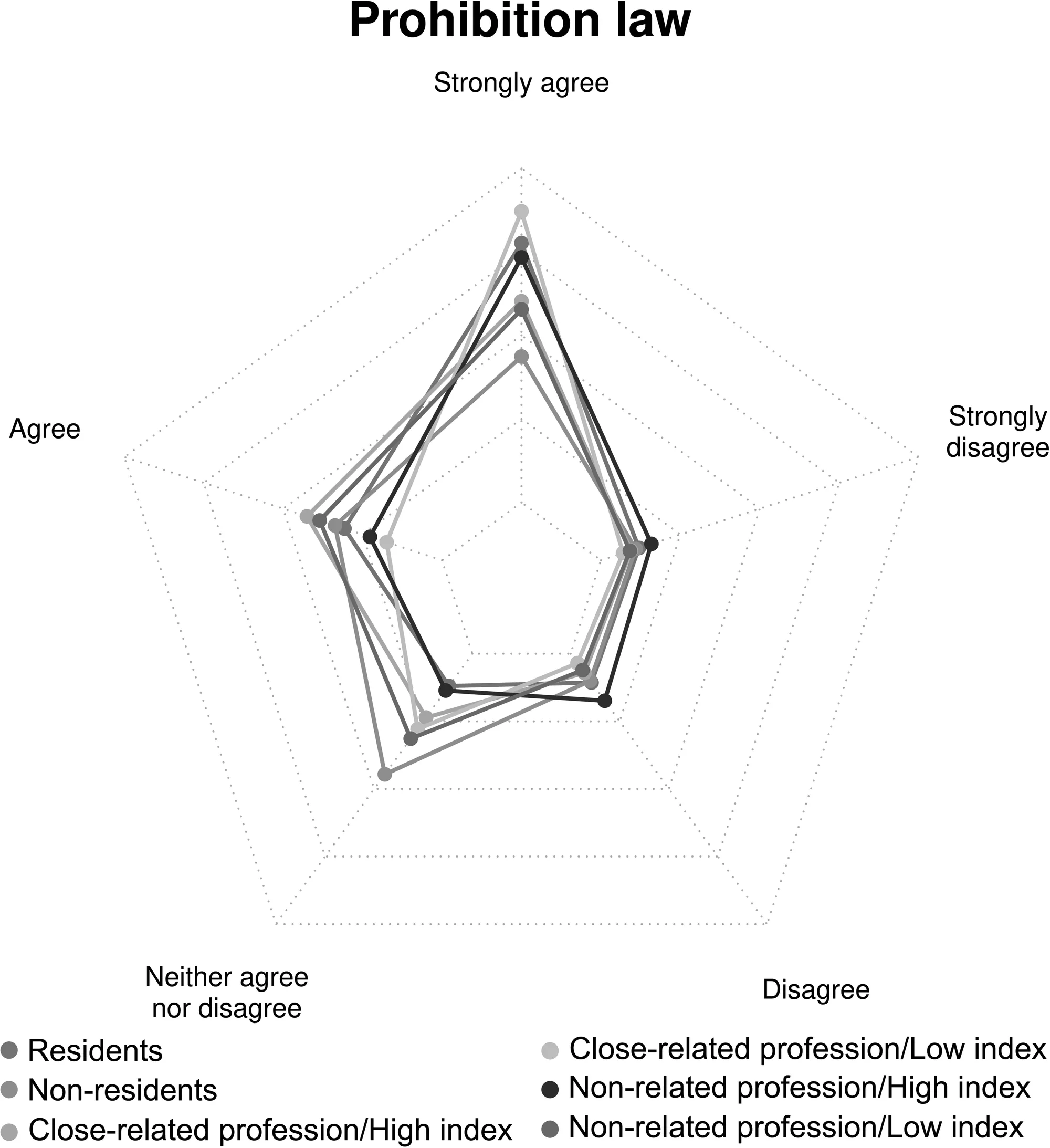
Radar chart displaying respondents’ perceptions across the question included in Section 5: Prohibition law. A brief description of each question, along with its corresponding line color, is provided. Figure 6. long description.

Strong support was expressed for the recent provincial regulation prohibiting intensive salmonid aquaculture in TDF (Figure 6). Nearly 80% of TDF residents supported the law, with 55% indicating strong agreement (Figure 6A). Among nonresidents, the most frequent responses were either strong agreement, although to a lesser extent than among residents, or neutrality (Figure 6B). Across the interaction profiles, support for the law remained high, although respondents without a close-related profession and with a low knowledge index (Figure 6F) showed a greater tendency toward neutral responses, whereas stronger agreement was more evident among the remaining combinations (Figure 6C–E).

## Discussion

Previous studies have well recognized the diversity and often conflicting nature of public opinion regarding trout and salmon aquaculture in their native and non-native distribution regions. For instance, Condie et al. (2022), analyzing perspectives across multiple stakeholder groups in Australia, found sharp divergences between pro-industry actors who emphasize innovation and sustainability, and community members who associate aquaculture with marine pollution, inadequate communication and governance. Similarly, Weitzman et al. (2022) highlighted that sociodemographic characteristics shape opinions in Nova Scotia, Canada: rural communities may oppose salmon farming due to its perceived threats to wild stocks and water quality, while some urban populations see it as a solution for food security. However, Hynes et al. (2018) demonstrated that while aquaculture is perceived as a significant industry in Ireland, public concern about its impacts is notably higher there compared to Norway, where aquaculture is deeply established and ranks as the second-largest industry after oil. Collectively, these studies emphasize that public opinion is shaped by a complex interplay of context-specific values, trust in governance institutions and access to information, factors that make achieving consensus on salmonid aquaculture particularly challenging (Young et al., 2019). Our study hypothesized that residence, level of knowledge and professional background would explain variability in public perceptions of salmonid aquaculture in southern Patagonia. Differences were found between residents and nonresidents. While the underlying causes were not directly investigated, prior research suggests that residents often express stronger environmental concern due to the proximity and potential impact on their own place of residence (Shafer et al., 2010; Van Putten et al., 2018). Thus, high levels of place attachment (*i.e.*, the emotional and cognitive bond with a specific environment (Williams and Roggenbuck, 1989) may help explain strong local resistance to new aquaculture developments.

Other individual traits, shaped by cultural context, may also influence preferences for particular environmental conditions, driven by personal experience, engagement and commitment. This perspective may help explain the response patterns associated with the interaction between profession relatedness to natural sciences and knowledge level (low or high). Additionally, the intense public discourse that preceded the passing of the prohibition law, including media coverage, public demonstrations, gastronomic events and outreach activities, likely contributed to increased awareness and opposition among the local population. This interpretation is supported by our media content analysis, which showed a greater number of articles and a higher density of opposition-related terminology in local media, particularly aligned with the “No to Salmon Aquaculture” movement.

Despite the overall heterogeneity of responses, some shared opinion patterns emerged. Most notably, there was strong opposition to net-pen aquaculture of salmonids in the BC. Among the 388 respondents (79%) who opposed salmon aquaculture in net-pen, 91% also opposed trout aquaculture. However, acceptance increased when aquaculture was proposed in land-based systems: only 30% and 21% opposed land-based salmon and trout farming, respectively. These results indicate that concern is primarily directed at ecological impacts on the BC, including organic waste discharge, escape of exotic species, unpredictable interactions with native biodiversity and antibiotic use, all of which have been documented in the Chilean salmon farming industry (Buschmann et al., 2009; Sepúlveda et al., 2013; Quiñones et al., 2019; Rodríguez-Luna et al., 2021). From a territorial perspective, García et al. (2020) identified governance gaps in regulating intensive salmon aquaculture in the BC, including limited enforcement capacity, underdeveloped value chains and low employment benefits. These limitations emerged partially among the respondents. While respondents tended to agree that the proposed aquaculture developments could generate economic benefits, the high frequency of neutral responses suggests uncertainty or limited familiarity with their potential socioeconomic implications, rather than a clearly defined positive or negative position. Despite this, most respondents, particularly residents and respondents with professions related to natural sciences, supported the legal prohibition of intensive salmonid aquaculture. Indeed, following the 2021 passage of Provincial Law No. 1355, Argentina became the first country to ban intensive salmon aquaculture at the provincial level. The law’s first article prohibits salmonid aquaculture in all lacustrine and marine waters of TDF, except for stocking activities overseen by a designated authority. This exception highlights that salmonids still hold local value as biological resources, particularly in the context of repopulation programs.

Salmonids were introduced to TDF in 1931 (Macchi and Vigliano, 2014), and today only brown trout, rainbow trout and brook trout have established feral populations. As a result, recreational trout fishing has become an economically and culturally significant activity (O’Neal and Stanford, 2011; Casalinuovo et al., 2017), although its precise economic contribution remains unquantified. The long-standing presence of trout in Argentina and its cultural relevance may explain public acceptance of maintaining already established exotic populations (García de Leaniz et al., 2010). By contrast, the introduction of Atlantic salmon for aquaculture development received no support. Though Chinook and coho salmon have been recorded since 2009 and 2019, respectively (Fernández et al., 2010; Chalde et al., 2019), public perception remains critical. Almost no respondents reported positive ecological impacts from either trout or salmon, and negative effects were more frequently attributed to salmon. This could reflect both a lack of awareness of trout’s status as a salmonid and a greater sense of acceptance or familiarity due to its longer regional presence. No strong support emerged for the eradication of established salmonids, reinforcing the idea that the general public views their impact as ecologically neutral, particularly in the case of trout.

The debate over exotic species in TDF is not unique to salmonids; it echoes a broader, historically contentious narrative involving invasive mammals and plants (Anderson and Valenzuela, 2014). However, recent efforts to establish salmonid aquaculture in the BC have reignited this controversy, highlighting its nature as a *wicked problem* sensu Jentoft and Chuenpagdee (2009). Such problems defy simple solutions because they are rooted in deep social conflicts, scientific uncertainty and multiple, often incompatible, stakeholder values. Our findings suggest that public opinion surrounding salmonids in TDF cannot be reduced to a simplistic binary of economic development *versus* environmental protection. Instead, perceptions are context-dependent and nuanced. While some forms of salmonid presence, such as food in restaurants and recreational trout fishing are generally tolerated or even valued by local residents and tourists, other practices, particularly intensive net-pen farming of Atlantic salmon in ecologically sensitive or symbolically significant areas, trigger strong opposition. This variability highlights the need to approach salmonid aquaculture governance with a more flexible and deliberate framework. The divergence in stakeholder postures reflects differing priorities, knowledge and definitions of sustainability. Hence, addressing the issue demands not just technical or regulatory solutions, but processes that engage with the social dimensions of the conflict, including local and transnational knowledge, cultural values and legitimacy concerns. Moreover, the tourism sector plays a critical role in this debate as an economic pillar of TDF. Nature-based tourism oriented toward marine environments, including activities such as marine wildlife watching (*e.g.*, penguins, sea lions and whales), kayaking, coastal hiking and interpretive boat tours through the BC, is tightly coupled with the perception of an unspoiled environment (Tartaglia Gamarra et al., 2026). The visual and ecological impacts of large-scale aquaculture infrastructure are seen as incompatible with the region’s ecotourism brand and long-term development model (Campolunghi et al., 2025).

### Contradictory social and governance valuation of exotic salmonids in Patagonia

Regional management has long combined two partially conflicting logics: the conservation of native fish and the promotion of exotic salmonids as valuable resources for recreational fisheries and associated local economies (Pascual et al., 2009; Llompart et al., 2024). In Patagonia, this has historically involved stocking, hatchery support and fishing regulations that helped normalize trout as socially desirable fish, even when their ecological effects were increasingly recognized (Cussac et al., 2016). In TDF, salmonids have long been associated with recreational fisheries and stocking (O’Neal and Stanford, 2011; Casalinuovo et al., 2017), whereas their use for open-water aquaculture remains novel and socially unfamiliar. This difference in historical presence may be important, as social acceptance tends to be lower for activities that lack a long local trajectory and remain publicly associated with uncertainty and risk (Kraly et al., 2022). Indeed, García de Leaniz et al. (2010) argued that salmonids in the southern hemisphere may be viewed as either beneficial or harmful depending on where they occur, how they were introduced and what benefits they provide. This helps explain why respondents tended to perceive salmon as more ecologically harmful than trout yet also showed high disagreement with eradication. Such responses do not necessarily indicate inconsistency but rather reflect that trout have become culturally familiar and institutionally tolerated in Patagonia. However, in southern Chile, trout dominate fish assemblages across broad areas and are strongly associated with reductions in native fish occurrence and abundance (Soto et al., 2006). More recently, Soto et al. (2023) showed that rainbow trout may represent higher long-term escape risk than Atlantic salmon because of its greater survival, trophic impact and capacity to establish self-sustaining populations in the wild; importantly, trout propagation linked to recreational fisheries remains common on the Argentine side of Patagonia. Thus, the debate in TDF should not focus only on Atlantic salmon farming in net-open, but also on the broader contradiction by which salmonids may be simultaneously valued, propagated, tolerated and rejected depending on context. A more coherent governance approach would require connecting aquaculture, stocking, recreational fisheries, farming and biodiversity conservation within the same policy framework.

In this context, the conflict surrounding salmon farming is not only about species or production methods, but also about legitimacy, local identity and socially acceptable pathways for coastal development. From a social license to operate perspective (Ford et al., 2022), our results suggest that intensive salmonid aquaculture, particularly in the form of net-pen farming in the BC, currently lacks broad social acceptance. Although our study does not allow causal inference about the policy process itself, the strong support observed for Provincial Law No. 1355 is consistent with the view that broad social opposition formed part of the political and institutional context in which the law was adopted.

## Conclusion

This study shows that public perceptions of exotic salmonid aquaculture in TDF are not simply polarized between economic development and environmental protection, but are shaped by residence, professional background, knowledge and the historical role of salmonids in Patagonia. Respondents strongly rejected open net-pen salmonid aquaculture in the BC, particularly for Atlantic salmon, while showing greater acceptance of land-based systems, native fish aquaculture and the continued presence of already established trout populations. Importantly, broad support for Provincial Law No. 1355 was observed across respondent profiles, indicating that the prohibition of intensive salmonid aquaculture in the BC has strong social legitimacy in the local context. In TDF, governance should distinguish between species, production systems, pathways of introduction and management purposes, while integrating aquaculture regulation, recreational fisheries, stocking practices, biodiversity conservation and socioeconomic priorities into a coherent framework. The latter requires clearer public communication not only for technical or economic feasibility, but also for ecological risk, territorial identity and social acceptability.

## Supporting information

10.1017/cft.2026.10045.sm001 Llompart et al. supplementary material 1 Llompart et al. supplementary material

10.1017/cft.2026.10045.sm002 Llompart et al. supplementary material 2 Llompart et al. supplementary material

## Data Availability

Data available on request from the authors.

## Footnotes

1
https://www.argentina.gob.ar/ciencia/argentina-innovadora-2030/plan-argentina-innovadora-2020.

2
http://www.agencia.mincyt.gob.ar/upload/Bases%20y%20Condiciones%20INNOVACUA%202016_final.pdf.

## Supplementary material

The supplementary material for this article can be found at http://doi.org/10.1017/cft.2026.10045.

## Long descriptions

### Figure 1. Long description

A scatter plot with Factor 1 on the x-axis (43.95 percent) and Factor 2 on the y-axis (30.71 percent). The data is organized into four color-coded word clusters across the quadrants.

* Top-Left Quadrant (Cyan): Concentrated between Factor 1 values of -2 to 0 and Factor 2 values of 0 to 2. Prominent words include ley, prohibir, legislatura, tierra, and proyecto.

* Top-Right Quadrant (Purple): Concentrated between Factor 1 values of 1 to 3 and Factor 2 values of 1 to 4. Prominent words include lobo, alga, marino, enfermedad, and investigador. Smaller words like pez and antibiótico appear closer to the center.

* Bottom-Right Quadrant (Red): Concentrated between Factor 1 values of 0 to 2 and Factor 2 values of -2 to 0. Prominent words include salmón, pescado, carne, millón, and más.

* Bottom-Left Quadrant (Green): Concentrated between Factor 1 values of -2 to 0 and Factor 2 values of -2 to 0. Prominent words include gobierno, información, productivo, and modelo.

The axes range from -3 to 4 on the y-axis and -2 to 3 on the x-axis, with dashed lines intersecting at the zero origin to define the four zones.

Navigate back to Figure 1..

### Table 1. Long description

The table consists of six columns: Factors, d f, M S, Pseudo-F, p (perm), and Unique perm.

* Residence (R): d f 1, M S 0.20, Pseudo-F 7.45, p (perm) 0.003, Unique perm 9,987.

* Profession (P): d f 1, M S 0.66, Pseudo-F 24.59, p (perm) 0.0001, Unique perm 9,969.

* Index (I): d f 1, M S 0.23, Pseudo-F 8.64, p (perm) 0.002, Unique perm 9,982.

* R x P: d f 1, M S 0.05, Pseudo-F 2.19, p (perm) 0.16, Unique perm 9,974.

* R x I: d f 1, M S 0.09, Pseudo-F 3.49, p (perm) 0.07, Unique perm 9,979.

* P x I: d f 1, M S 0.12, Pseudo-F 4.75, p (perm) 0.02, Unique perm 9,966.

* R x P x I: d f 1, M S 0.06, Pseudo-F 2.46, p (perm) 0.12, Unique perm 9,969.

* Residual: d f 484, M S 0.02.

* Total: d f 491.

Navigate back to Table 1..

### Table 2. Long description

The table consists of four columns: Group index, t, p open parenthesis perm close parenthesis, and Unique perms.

- The first section is titled Within level close of factor Profession. It contains one row: Group index High, low; t value 1.7221; p open parenthesis perm close parenthesis 0.0917; and 9,988 unique perms.

- The second section is titled Within level no-close of factor Profession. It contains one row: Group index High, low; t value 3.6334; p open parenthesis perm close parenthesis 0.0001; and 9,977 unique perms.

A footer note specifies that Student’s t values, permutation-based p-values, and the number of unique permutations are displayed.

Navigate back to Table 2..

### Figure 2. Long description

A multi-panel figure with six radar charts labeled A through F. Each chart uses a pentagonal grid with five axes representing levels of agreement: Strongly agree at the top, followed clockwise by Strongly disagree, Disagree, Neither agree nor disagree, and Agree. Seven colored lines represent different aquaculture types: Salmon in net pen in the B C (light blue), Salmon in I M T A in the B C (cyan), Salmon on land-based system (brown), Trout in net pen in the B C (pink), Trout in I M T A in the B C (green), Trout on land-based system (orange), and Native fish in the B C (olive).

* Panel A (Residents) and Panel B (Non-residents): Show relatively balanced perceptions with slight peaks toward Agree for land-based and I M T A systems.

* Panel C (Close-related profession/High index) and Panel D (Close-related profession/Low index): Panel D shows a sharp spike toward Strongly disagree for salmon and trout in net pens.

* Panel E (Non-related profession/High index) and Panel F (Non-related profession/Low index): Show moderate agreement for native fish and land-based systems, while net pen systems trend toward the center or disagree axes.

Navigate back to Figure 2..

### Figure 3. Long description

The figure consists of six panels labeled A through F, each featuring a pentagonal radar chart with five axes representing levels of agreement: Strongly agree (top), Strongly disagree (top-right), Disagree (bottom-right), Neither agree nor disagree (bottom-left), and Agree (top-left).

* Panel A: Residents.

* Panel B: Non-residents.

* Panel C: Close-related profession/High index.

* Panel D: Close-related profession/Low index.

* Panel E: Non-related profession/High index.

* Panel F: Non-related profession/Low index.

Each chart contains six colored lines representing different interaction types:

* Light blue: Salmon interaction in freshwater.

* Pink: Trout interaction in freshwater.

* Brown: Salmon alter water conditions.

* Dark blue: Salmon interaction in the sea.

* Green: Trout interaction in the sea.

* Orange: Trout alter water conditions.

Across all panels, the data points are concentrated between the center and the Neither agree nor disagree or Disagree axes, indicating a general lack of strong agreement with the statements. Panels C and D show a slightly wider spread toward the Strongly disagree axis compared to the other groups.

Navigate back to Figure 3..

### Figure 4. Long description

The figure consists of six panels labeled A through F. Each panel is a pentagonal radar chart with five axes representing levels of agreement: Strongly agree at the top, followed clockwise by Strongly disagree, Disagree, Neither agree nor disagree, and Agree.

Five variables are plotted in each chart:

* Salmon eradication (light blue)

* Salmon eradication in protected areas (dark blue)

* Trout eradication (pink)

* Trout eradication in protected areas (green)

* Introduction of new salmon and trout in T D F (brown)

Panel A (Residents) and Panel B (Non-residents) show similar patterns where most eradication variables cluster between Agree and Neither agree nor disagree, while the introduction of new species (brown line) leans more toward Strongly agree.

Panel C (Close-related profession/High index) and Panel D (Close-related profession/Low index) show that the High index group has a tighter consensus toward Agree for eradication, while the Low index group shows more variance, particularly with trout eradication in protected areas extending toward Neither agree nor disagree.

Panel E (Non-related profession/High index) and Panel F (Non-related profession/Low index) display the widest variance. In Panel F, the green and blue lines for eradication in protected areas stretch significantly toward the Neither agree nor disagree and Disagree axes compared to the High index group in Panel E.

Navigate back to Figure 4..

### Figure 5. Long description

The figure consists of six panels labeled A through F, each featuring a pentagonal radar chart. The five axes represent a Likert scale: Strongly agree at the top, moving clockwise to Strongly disagree, Disagree, Neither agree nor disagree, and Agree. Two data series are plotted: a blue line for ‘Salmon and trout aquaculture are economically relevant for T D F’ and a pink line for ‘Prohibition of salmon and trout in restaurants’.

* Panel A, Residents: Blue line peaks at Agree. Pink line peaks at Disagree.

* Panel B, Non-residents: Blue line peaks between Agree and Strongly agree. Pink line peaks at Disagree.

* Panel C, Close-related profession/High index: Blue line is concentrated near Agree. Pink line shows a wide spread toward Disagree and Strongly disagree.

* Panel D, Close-related profession/Low index: Blue line peaks at Agree. Pink line is centered around Neither agree nor disagree and Disagree.

* Panel E, Non-related profession/High index: Blue line peaks at Agree. Pink line peaks at Strongly disagree.

* Panel F, Non-related profession/Low index: Blue line peaks between Agree and Strongly agree. Pink line peaks at Strongly disagree.

Across all panels, the blue series generally trends toward agreement regarding economic relevance, while the pink series trends toward disagreement regarding restaurant prohibition.

Navigate back to Figure 5..

### Figure 6. Long description

The radar chart features five axes arranged in a pentagon. Starting from the top and moving clockwise, the axes are labeled: Strongly agree, Strongly disagree, Disagree, Neither agree nor disagree, and Agree. Six colored lines represent different respondent groups: Residents (gray), Non-residents (pink), Close-related profession/High index (blue), Close-related profession/Low index (green), Non-related profession/High index (brown), and Non-related profession/Low index (orange).

Data distribution shows:

* Strongly agree: The green line (Close-related profession/Low index) reaches the highest point near the outer perimeter, followed by gray, blue, brown, orange, and pink.

* Strongly disagree: All groups are clustered near the center, indicating low frequency for this response.

* Disagree: Responses are slightly higher than strongly disagree but remain concentrated toward the inner core.

* Neither agree nor disagree: The pink line (Non-residents) extends furthest toward the perimeter, followed by orange and green.

* Agree: The blue line (Close-related profession/High index) and gray line (Residents) show the highest values on this axis.

Overall, the chart shows a strong skew toward the Strongly agree and Agree axes for most groups, with Non-residents showing a higher tendency toward neutral responses.

Navigate back to Figure 6..
