# Supplementary material for: Social attitude toward exotic salmonids aquaculture in the Beagle Channel, Argentina: Productive opportunity and/or conservation concerns?
Source: Camb Prism Coast Futur. 2026 Sep 9;4:e26. doi: 10.1017/cft.2026.10045 (PMC13748896; doi:10.1017/cft.2026.10045)
Supplement: Supplementary file 1 [file S2754720526100456sup001.docx]

**Title:** Social attitude towards exotic salmonids aquaculture in the Beagle Channel, Argentina: productive opportunity and/or conservation concerns?

**Authors:** Facundo Llompart, Javier H. Rojo, Silvina A. Romano, Tomás Chalde

**Supplementary Material 1:**

Structure of the online survey conducted using the Google Forms platform between July 19th and September 8th, 2021

**General questions**: Identification of the interviewee's profile.

a) Place of residence

- Tierra del Fuego Province
- Other

b) Highest level of studies achieved; specify title obtained.

- No studies
- Primary school
- Secondary school
- Tertiary school
- University
- Postgraduate

c) Do you belong to an NGO related to environmental protection?
d) Do you belong to a recreational fishing association?
e) Are you a recreational trout or salmon fisher?
f) Indicate True, False, or I do not know.
1. Trout and salmon are native to Patagonia.
2. All the trout and salmon migrate to the sea and return to the river to reproduce.
3. There are no salmon in Tierra del Fuego.
4. The only fish that inhabit Tierra del Fuego 's lakes, rivers, and streams are trout and salmon.
5. The trout come from escapes from Chilean salmon farming.

**Section 1. Aquaculture:** Aquaculture practices involving salmonids and native species in TDF across various production systems.

What is your opinion regarding the following activities related to trout and salmon farming at Tierra del Fuego? Strongly agree, agree, neither agree nor disagree, disagree, strongly disagree.

**Q1.** Salmon farming in cages in the Beagle Channel for human consumption.

**Q2.** Trout farming in cages in the Beagle Channel for human consumption.

**Q3.** Salmon farming in multitrophic farms in the Beagle Channel for human consumption.

**Q4.** Trout farming in multitrophic farms in the Beagle Channel for human consumption.

**Q5.** Salmon farming on land-based systems for human consumption.

**Q6.** Trout farming on land-based systems for human consumption.

**Q7.** Native fish farming in the Beagle Channel for human consumption.

**Section 2. Salmonids interactions:** Ecological interactions between salmonids and other fish species, including their environmental impact.

How do you think trout and salmon affect the environment? Strongly agree, agree, neither agree nor disagree, disagree, strongly disagree.

**Q8.** Salmon interact with other fish in rivers, streams, and lakes.

**Q9.** Trout interact with other fish in rivers, streams, and lakes.

**Q10.** Salmon interact with other marine fish.

**Q11.** Trout interact with other marine fish.

**Q12.** Salmon modify water quality.

**Q13.** Trout modify water quality.

**Section 3. Salmonids eradication:** Implementation of salmonid eradication measures.

Express your appreciation regarding the following socio-economic impacts related to trout and salmon. Strongly agree, agree, neither agree nor disagree, disagree, strongly disagree.

**Q14.** Salmon must be eradicated from TDF.

**Q15.** Trout must be eradicated from TDF.

**Q16.** Salmon must be eradicated only in protected areas, reserves, and the TDF National Park.

**Q17.** The introduction of new species of trout and salmon must be prohibited, but those that are already in TDF must be left.

**Q18.** If trout and salmon generate an environmental impact, the economic benefits provided by sport fishing and tourism compensate for it.

**Section 4: Salmonids economic impacts:** Economic importance of salmonids for TDF.

Express your appreciation regarding the following economic aspects. Strongly agree, agree, neither agree nor disagree, disagree, strongly disagree.

**Q19.** Trout and salmon aquaculture would be an important activity for the economy of Tierra del Fuego.

**Q20.** Trout and salmon must be prohibited in restaurants, fishmongers, and canned at TDF.

**Section 5: Prohibition Law:** Acceptance of Provincial Law 1355/21.

Express your level of agreement or disagreement with the following aspect. Strongly agree, agree, neither agree nor disagree, disagree, strongly disagree.

**Q21.** The recent Law 1355/21, enacted in TDF, relates to salmon farming.
