# Supplementary material for: Social attitude toward exotic salmonids aquaculture in the Beagle Channel, Argentina: Productive opportunity and/or conservation concerns?
Source: Camb Prism Coast Futur. 2026 Sep 9;4:e26. doi: 10.1017/cft.2026.10045 (PMC13748896; doi:10.1017/cft.2026.10045)
Supplement: Supplementary file 2 [file S2754720526100456sup002.docx]

**Title:** Social attitude towards exotic salmonids aquaculture the Beagle Channel, Argentina

**Authors:** Facundo Llompart, Javier H. Rojo, Tomás Chalde, Silvina A. Romano

**Supplementary Material 2:**

Main words from the National and Local media press detected from Corpus analysis applying the Alceste method (Reinert, 1987).

Group 1 refers to the promulgation of the law and the prohibits of the activity (prohibit, law, legislature).

Group 2 refers to conservation and biodiversity.

Group 3 refers to government (government, information).

Group 4 refers to the productive activity (salmon, millions).

| **Group 1** | **Group 2** | **Group 3** | **Group 4** |
| --- | --- | --- | --- |
| Law | Marine | Government | Salmon |
| Prohibit | Sea-lion | Study | More |
| Legislative | Algae | Information | Millions |
| Land | Fish | Signature | Fish |
| Project | Chemical | Productive | Do |
| Submit | Gustavo | Now | Meet |
| Fuego | Toxic | Model | One |
| Province | Disease | Secretary | Arrive |
| Legislator | Bottom | Job | Year |
| Provincial | Researcher | Advance | Restaurant |
| Resource | Crustacean | Development | Ton |
| Protection | Tide | Bertone | Artisanal |
| Lacustrine | Spread | Agroindustry | Consumption |
| Prohibition | Mammal | Agreement | Marine |
| Approve | Parasite | Charge | Harvest |
| Jurisdictional | Microalgae | Job | Thousand |
| Contemplate | Kill | Norway | Lake |

Reinert M. (1987). Classification descendante hiérarchique et analyse lexicale par contexte: application au corpus des poésies d'Arthur Rimbaud. *Bulletin Méthodologie Sociologique* **13**, 53–90.
