# Supplementary material for: Social attitude toward exotic salmonids aquaculture in the Beagle Channel, Argentina: Productive opportunity and/or conservation concerns?
Source: Camb Prism Coast Futur. 2026 Sep 9;4:e26. doi: 10.1017/cft.2026.10045 (PMC13748896; doi:10.1017/cft.2026.10045)
Supplement: Peer review history

# Reviewer report: Review: Social attitude toward exotic salmonids aquaculture in the Beagle Channel, Argentina: Productive opportunity and/or conservation concerns? — R0/PR2

**DOI:** 10.1017/cft.2026.10045.pr2

**Authors:** Suzannah-Lynn Billing

**Affiliations:** Scottish Association for Marine Science

Thank you to the authors for conducting and writing an interesting piece of research. I believe that it contributes to literature on social perceptions of aquaculture. It is particularly welcome as this topic seems to have few studies from South American countries.

I think it could contribute significantly more than it currently does, by situating the results within theory. This could be social acceptability theory, social license to operate, legitimacy– there’s quite a few to choose from. The reason I suggest this approach is that the results have clear links to these well-established and evidenced theories. It would be novel to be able to conclude that these theories apply to somewhere which does not currently (and probably won’t because of the prohibition) have extensive salmonid aquaculture. It strengthens the arguments of various previous studies, that social acceptability is a pillar of aquaculture operations in countries with engaged citizens and marine spatial planning (or similar) governance structures. To this end, this paper would benefit from wider reading on the social perceptions of aquaculture. There is quite a lot out there, that would support the arguments presented.

I suggest that more could be gleaned from the results of the survey through the use of more robust statistical analysis. The sample size (although the sampling strategy is not stated) is more than ample to do statistical comparisons, which would support statements of significance and relationships between “profiles” and perceptions. This would improve the robustness of the conclusions.

The discussion section could be improved by reflecting the recommendations provided above. It is currently missing references to back up some of the arguments presented.

Lastly, a note on style. For my preferences, there is over-use of the em dash and the term “underscores” (as I sometimes find in LLM generated text. Although not after very recent updates). Less frequent use of both might improve the flow of the writing. There are some typos to check for, but overall, it reads well.

Below are some suggestions per section.

Introduction: this section is under-referenced (as is the discussion section). There is quite a lot of work out there. Recommendations include but are not limited to: Krause et al., 2015 10.1016/j.aquaculture.2015.02.009 , Agúndez et al., 2025 10.1093/icesjms/fsaf100, Osmundsen et al., 2017 10.1080/13657305.2017.1262476, Mather and Fanning 2019, 10.1016/J.MARPOL.2018.10.049, Billing, 2018 10.1016/j.ocecoaman.2018.09.011

Line 67: suggest removing comparison to fishing, it complicates the opening sentence

Line 79: references needed for this sentence

Line 82: remove “even”

Line 90 and throughout: “net-pen” is the more commonly used technical term for fish farming in marine environments. Suggest changing to “net-pen” throughout.

Line 111: “driven by public opposition” - this requires references.

Line 112: full reference for the provincial law required.

Materials and Methods: This section requires more information on choice of methods, supporting arguments for choice of methods and references to support the reasoning. E.g. why did you choose Press corpus analysis? Why did you choose 2018-2021? Why did you choose an online survey over other techniques? Why did you design it the way you did? Where you inspired by other papers or methods books? Why did you choose a knowledge index? Why did you design the knowledge index the way that you did? Why did you choose the “profiles” that you have included and not others? Why did you choose the distribution methods you have? What sampling strategy did you use? Why did you choose to not conduct statistical analysis? What is your rational for descriptive statistics?

Line 176: include “neutral” as you list the rest of the options.

Results: I think this is probably an issue of formatting and may be a result of journal requirements; I found it tricky to navigate the figures and flip between the end of the document and the analysis section. If possible, would recommend placing the figures in a location near the text describing them. The figure captions don’t really match with what’s being described in the text either. It is decipherable, but I think could be improved.

Line 204 – 205: Any reasons for these results? Would be nice to see some commentary (e.g. does this reflect population demographics or is it a result of your sampling strategy?).

Line 214: “The knowledge index did not influence the perception about salmonid aquaculture in TDF.” Without conducting statistics, I’m not sure you can derive a conclusion like this about the relationship between these two variables.

Discussion: This section is under-referenced. Would suggest reading around theory and other aquaculture perception studies (e.g. those listed above for a start). Inclusion of other evidence will strengthen your arguments.

Line 295: are there any RAS systems currently in operation? I wonder whether knowledge (or lack thereof) influences this result?

Line 305: Is it ambivalence or is it less knowledge? Earlier you suggest the latter.

Line 318: would remove “... new exotic species such as the one proposed for the development of aquaculture...” It’s a bit wordy, the reader knows you’re talking about salmonids.

Line 324: references needed around exposure and establishment of production systems and social acceptability; there is work out there on this.

Line 335: “...perceptions are context-dependent and nuanced” is a well-evidenced in social license to operate theory. Could include more refs here to support this argument.

Line 434: Introducing tourism here is a bit odd as it’s not in the introduction. Would add a paragraph on this context in the introduction as it seems critical to opinions. And include references.

Line 347: Is evidence that says there are perceptions of unspoilt environments? Or is this an assumption?

Line 348: Visual impact should also be included in the introduction if you want to include it here. There is a raft of research on visual impact, worth considering if you want to make this point.

Line 350: Regional identity clashes between salmon aquaculture and local communities have been recorded by Ford et al (2022 - 10.1016/j.aquaculture.2022.738081) and probably others. Sustainability concerns are well documented across aquaculture literature. Worthwhile pointing to these sources and perhaps introduce these concepts in the introduction.

Line 351: I wonder at who in your study is ignoring social complexity? To me, your study reads as if public opinion has influenced policy directly resulting in the prohibition – which is a very interesting case.

Line 353: Your survey doesn’t really present evidence related to place-based values, local resistance or reputational damage. My suggestion is that you present a concluding sentence about your interesting findings, rather than evidence from other sources (which are not referenced).

# Reviewer report: Review: Social attitude toward exotic salmonids aquaculture in the Beagle Channel, Argentina: Productive opportunity and/or conservation concerns? — R0/PR3

**DOI:** 10.1017/cft.2026.10045.pr3

**Authors:** Doris Soto

**Affiliations:** Universidad de Concepcion, Chile

Interestingly respondents do not see trout negative interactions in rivers and the sea as relevant while they see salmon negative interactions as very relevant (line 322 and Figures). However, when the question refers to trout or salmon eradication disagreement is high. These responses can be explained by the” bipolar” governance and social perception of salmonids in Patagonia. On the one hand, both in Chile and Argentina wild exotic salmonid are considered a resource for recreational fisheries, specially trout but also more recently chinook salmon (notice the recreational fishery rules both in Argentina and Chile). On the other hand, farmed salmonids in cages are considered a threat, especially escapees. These facts probably influence the perception that trout does not have a negative impact as relevant as salmon (Figures). This also could explain the wide disagreement with salmonid eradication and prohibition of salmon and trout from restaurants.

Probably most people do not know that trout poses a high risk for native biodiversity in rivers (Soto et al 2006) and caged trout escapes pose a higher risk than salmon (specially Atlantic salmon) escapes on biodiversity (See Soto et al 2022). Furthermore, trout are stocked on natural ecosystems for recreational purposes in TDF (and stocking comes from aquaculture), and this could be as or more important threat than trout and salmon escapes from cages !!. Therefore, the debate on farming exotic species should carefully analyze the contradictory protection for the same species or their relatives for recreational fisheries in TDF.

I think the above issues should be explored further in the discussion even with a specific heading because it could help authors to understand answers and, more importantly, provide recommendations regarding the need to better communicate scientific information regarding aquaculture and recreational fisheries with exotic species.

Cited references

Soto, D., I. Arismendi, J. Gonzalez, E. Guzman, J. Sanzana, F. Jara, C. Jara and A. Lara. 2006. Southern Chile, trout and salmon country: invasion patterns and threats for native species Rev. Chi. Natural History, 79: 97-117.

Soto, D, Arismendi I, Olivos, A., Canales, C, Leon-Muñoz, J., , Niklitscheck E., Paredes F., Sepulveda M., Gomez-Uchida, D., Soria-Galvarro Y. Environmental risk assessment of non-native salmonid escapes from net pens in the Chilean Patagonia. 2022. Reviews in Aquaculture. https://doi: 10.1111/raq.12711

# Editor report: Recommendation: Social attitude toward exotic salmonids aquaculture in the Beagle Channel, Argentina: Productive opportunity and/or conservation concerns? — R0/PR4

**DOI:** 10.1017/cft.2026.10045.pr4

**Affiliations:** Institute for Coastal and Marine Research, Nelson Mandela University, South Africa

Dear authors 

Thank-you for your interesting contribution; please address the reviewers’ input (one major, one minor revision).

The reviewer would like you to investigate and apply “social acceptability theory”, consider some statistical analysis and use references to back up arguments in the Discussion and details in the Introduction. The Methods section requires additional detail on choice of methods. Highlight the important finding that public opinion influenced policy resulting in prohibition. Conclude with this as a finding rather than evidence from other sources.

Reviewer 2 would like to see a section in the Discussion on “the debate on farming exotic species should carefully analyze the contradictory protection for the same species or their relatives for recreational fisheries in TDF”.

# Editor report: Decision: Social attitude toward exotic salmonids aquaculture in the Beagle Channel, Argentina: Productive opportunity and/or conservation concerns? — R0/PR5

**DOI:** 10.1017/cft.2026.10045.pr5

**Affiliations:** Geography, Cambridge University, United Kingdom of Great Britain and Northern Ireland

No accompanying comment.

# Reviewer report: Review: Social attitude toward exotic salmonids aquaculture in the Beagle Channel, Argentina: Productive opportunity and/or conservation concerns? — R1/PR7

**DOI:** 10.1017/cft.2026.10045.pr7

**Authors:** Suzannah-Lynn Billing

**Affiliations:** Scottish Association for Marine Science

Thank you to the authors for all of their hard work in addressing my review comments – particularly in conducting robust statistical analysis to support their arguments, and inclusion of more theory related to social perceptions of aquaculture.

# Editor report: Recommendation: Social attitude toward exotic salmonids aquaculture in the Beagle Channel, Argentina: Productive opportunity and/or conservation concerns? — R1/PR8

**DOI:** 10.1017/cft.2026.10045.pr8

**Affiliations:** Institute for Coastal and Marine Research, Nelson Mandela University, South Africa

Thank-you, the comments of Reviewer 1 are addressed. Additional literature has been added, and the discussion expanded. Social acceptability is well described as well as the novelty of a country without salmon aquaculture. The methods section has been expanded and statistics added. 

The additional references listed by Reviewer 1 have not been added but others have.

Krause et al., 2015 10.1016/j.aquaculture.2015.02.009

Agúndez et al., 2025 10.1093/icesjms/fsaf100

Osmundsen et al., 2017 10.1080/13657305.2017.1262476

Mather and Fanning 2019, 10.1016/J.MARPOL.2018.10.049

Billing, 2018 10.1016/j.ocecoaman.2018.09.011

The comments from Reviewer 2 were fully addressed. The Discussion was reorganized and a new subsection added to interpret findings.

# Editor report: Decision: Social attitude toward exotic salmonids aquaculture in the Beagle Channel, Argentina: Productive opportunity and/or conservation concerns? — R1/PR9

**DOI:** 10.1017/cft.2026.10045.pr9

**Affiliations:** Geography, Cambridge University, United Kingdom of Great Britain and Northern Ireland

No accompanying comment.
